# Low-Dose Antibiotic Prophylaxis Induces Rapid Modifications of the Gut Microbiota in Infants With Vesicoureteral Reflux

**DOI:** 10.3389/fped.2021.674716

**Published:** 2021-06-17

**Authors:** William Morello, Federica D'Amico, Jessica Serafinelli, Silvia Turroni, Isabella Abati, Jessica Fiori, Esra Baskin, Fatos Yalcinkaya, Augustina Jankauskiene, Marco Pennesi, Aleksandra Zurowska, Francesca Becherucci, Dorota Drozdz, Djalila Mekahli, Grazyna Krzemien, Claudio La Scola, Katarzyna Taranta-Janusz, Otto Mehls, Franz Schaefer, Marco Candela, Giovanni Montini

**Affiliations:** ^1^Pediatric Nephrology, Dialysis and Transplant Unit, Fondazione IRCCS Ca' Granda, Ospedale Maggiore Policlinico, Milan, Italy; ^2^Unit of Microbiome Science and Biotechnology, Department of Pharmacy and Biotechnology, University of Bologna, Bologna, Italy; ^3^Department of Chemistry “Giacomo Ciamician,” University of Bologna, Bologna, Italy; ^4^Department of Pediatric Nephrology, Baskent University Hospital, Ankara, Turkey; ^5^Division of Pediatric Nephrology, Department of Pediatrics, School of Medicine, Ankara University, Ankara, Turkey; ^6^Clinic of Children Diseases, Institute of Clinical Medicine, Vilnius University, Vilnius, Lithuania; ^7^Department of Pediatrics, Institute for Maternal and Child Health—IRCCS “Burlo Garofolo,” Trieste, Italy; ^8^Pediatric Nephrology Department, Medical University of Gdansk, Gdansk, Poland; ^9^Nephrology and Dialysis Unit, Meyer Children's Hospital, Florence, Italy; ^10^Department of Pediatric Nephrology, Jagiellonian University Medical College, Krakow, Poland; ^11^Department of Development and Regeneration, Laboratory of Pediatrics, PKD Group, KU Leuven—University of Leuven, Leuven, Belgium; ^12^Department of Pediatric Nephrology, University Hospitals Leuven, Leuven, Belgium; ^13^Department of Pediatric Nephrology, The Medical University of Warsaw, Warsaw, Poland; ^14^Nephrology and Dialysis Unit, Department of Pediatrics, Azienda Ospedaliero Universitaria Sant'Orsola-Malpighi, Bologna, Italy; ^15^Department of Pediatrics and Nephrology, Medical University of Bialystok, Bialystok, Poland; ^16^Division of Pediatric Nephrology, Center for Pediatrics and Adolescent Medicine, Heidelberg University Hospital, Heidelberg, Germany; ^17^Department of Clinical Sciences and Community Health, University of Milan, Milan, Italy

**Keywords:** gut microbiota, antibiotic prophylaxis, vesicoureteral reflux, urinary tract infection, children

## Abstract

**Background and Objectives:** Maturation of the gut microbiota (GM) in infants is critically affected by environmental factors, with potential long-lasting clinical consequences. Continuous low-dose antibiotic prophylaxis (CAP) is the standard of care for children with vesicoureteral reflux (VUR), in order to prevent recurrent urinary tract infections. We aimed to assess short-term GM modifications induced by CAP in infants.

**Methods:** We analyzed the GM structure in 87 infants (aged 1-5 months) with high-grade VUR, previously exposed or naïve to CAP. Microbial DNA was extracted from stool samples. GM profiling was achieved by 16S rRNA gene-based next-generation sequencing. Fecal levels of short- and branched-chain fatty acids were also assessed.

**Results:** 36/87 patients had been taking daily CAP for a median time of 47 days, while 51/87 had not. In all patients, the GM was predominantly composed by *Bifidobacteriaceae* and *Enterobacteriaceae*. Subgroup comparative analysis revealed alterations in the GM composition of CAP-exposed infants at phylum, family and genus level. CAP-exposed GM was enriched in members of *Enterobacteriaceae* and Bacteroidetes, especially in the genera *Bacteroides* and *Parabacteroides*, and showed a trend toward increased *Klebsiella*, often associated with antibiotic resistance. In contrast, the GM of non-CAP children was mostly enriched in *Bifidobacterium*. No differences were found in fatty acid levels.

**Conclusions:** In infants with VUR, even a short exposure to CAP definitely alters the GM composition, with increased relative abundance of opportunistic pathogens and decreased proportions of health-promoting taxa. Early low-dose antibiotic exposure might bear potential long-term clinical risks.

## Introduction

The gut microbiota (GM) is an individual-specific plethora of microorganisms, living in the gastrointestinal tract with a mutualistic relationship with the host and contributing to its homeostasis (1, 2). GM is essential for metabolic, immunologic, and neurological functions (3).

Established soon after birth, the GM describes a developmental trajectory during infancy, reaching an almost stable adult-like profile around 3 years of age (4, 5). In particular, the first months of life are a critical time window for GM maturation, during which various factors can affect its eubiotic state and trajectory, such as mode of delivery, formula or breast milk and, more importantly, the administration of drugs (6–8). These modifications may have short- and long-term consequences for health, as suggested by extensive literature (1, 2, 6, 7). Specifically, GM changes induced by early antibiotic exposure have been linked to weight gain and subsequent development of asthma, allergy, eczema, atopy, and inflammatory bowel disease (6, 7, 9, 10). Moreover, the GM of antibiotic-exposed infants can become an important reservoir of antibiotic-resistance genes, potentially leading to life-threatening complications (9, 11).

Although controversial, continuous antibiotic prophylaxis (CAP) administered at a sub-therapeutic daily dose, is currently considered the standard of care for infants with high-grade vesicoureteral reflux (VUR) in order to prevent recurrent urinary tract infections (UTIs), renal scarring, and chronic kidney damage (12–14). Moreover, antibiotic prophylaxis is recommended for various clinical conditions and altogether it may account for up to 28.6% of prescribed antibiotics in hospitalized children (15).

The ongoing randomized PREDICT trial was designed in the context of the controversial CAP cost-benefit ratio (EudraCT 2013-000309-21), aiming to clarify the efficacy of CAP in preventing the first symptomatic UTIs in infants with high-grade VUR. A nested study was planned to evaluate the GM trajectory during the first years of life, comparing patients randomized to CAP or not.

Here, we present a cross-sectional analysis of stool samples collected from 87 infants during the pre-trial screening phase, to assess the effect of the exposure to a sub-therapeutic dose of antibiotics on the GM composition in very young children.

## Methods

### Patient Selection

We performed a cross-sectional study, analyzing the GM composition and fecal levels of short- and branched-chain fatty acids (SCFAs and BCFAs, respectively) in a cohort of patients screened for the PREDICT trial from October 2014 to November 2018. The trial enrolls infants <5 months of age with high-grade VUR (III-V), without previous UTIs and with gestational age ≥ 35 weeks, who are randomized to prophylaxis or not and followed for 5 years. We identified all screened patients with a viable stool sample and available data regarding CAP exposure before randomization. Selected patients were divided in two groups: non-CAP vs. CAP patients (Supplementary Figure 1). The use of CAP before stool collection was only related to local practice and did not interfere with subsequent patient randomization. The long-term effect of CAP on the GM trajectories or UTIs is not object of this study.

The study was approved by the Institutional Review Board of all participating centers and written informed consent was provided by all parents or legal guardians (ethical approval code of the coordinating center: 106/2013/O/Sper).

### Samples and Data Collection

Stool samples were collected for each patient and immediately frozen at −80°C without processing. Samples were shipped in dry ice to the Department Pharmacy and Biotechnology, University of Bologna (Bologna, Italy) for GM and SCFA/BCFA analysis.

From the PREDICT trial database, data regarding patients (age, gender, VUR), previous antibiotic prophylaxis (agent, dose, and duration) and other potential GM-perturbing factors (feeding modality, delivery mode, gestational age, drug consumption, and probiotics use) were extracted.

### Microbial DNA Extraction, Library Preparation, and Sequencing

Microbial DNA was extracted from feces using the repeated bead-beating plus column method, as previously described (16). DNA concentration and quality were assessed with NanoDrop ND-100 (NanoDrop Technologies, Wilmington, DE, USA).

For library preparation, the V3-V4 hypervariable region of the 16S rRNA gene was amplified using 341F and 785R primers, as previously reported (16). PCR products were purified using a magnetic bead-based clean-up system (Agencourt AMPure XP, Beckman Coulter, Brea, CA, USA). A limited-cycle PCR was performed to obtain the indexed library using Nextera technology. Final libraries were sequenced on an Illumina MiSeq platform with a 2 x 250 bp paired-end protocol according to the manufacturer's instructions (Illumina, San Diego, CA, USA). Sequencing reads were deposited in the National Center for Biotechnology Information Sequence Read Archive (NCBI SRA; BioProject ID PRJNA706153).

### GC–MS Determination of Fecal SCFAs and BCFAs

Approximately 0.25 g of feces were analyzed for levels of SCFAs (acetic, propionic, butyric, and valeric acids) and BCFAs (isobutyric and isovaleric acids). Sample preparation was performed by head space-solid phase microextraction (HS-SPME), followed by gas chromatography–mass spectrometry (GC-MS) analysis, as previously described (17). The chromatogram acquisition was obtained with Total Ion Current (TIC) and Single Ion Monitoring (SIM) scan modes.

### Bioinformatics and Statistics

Between-group differences in patient characteristics were assessed using the Chi-square test or Wilcoxon test, to rule out potential confounders in the GM analysis.

For GM, raw sequences were processed using QIIME 2 (18). Length and quality-filtered reads were binned into amplicon sequence variants (ASVs) using DADA2 (19). Taxonomic assignment was carried out using the Greengenes database (May 2013 release). Chimeras were discarded during analysis. Alpha diversity was calculated using the number of observed ASVs and Faith's Phylogenetic Diversity, while beta diversity was estimated by computing UniFrac distances, which were used as input for Principal Coordinates Analysis (PCoA). All statistical analysis was performed using R (https://www.r-project.org/). PCoA plots were generated using the “vegan” and “Made4” packages, and data separation was tested by a permutation test with pseudo-F ratios (function “Adonis” in “vegan”). The bacterial genera most contributing to the ordination space were identified using the function “envfit” of “vegan”. Wilcoxon test was used to assess significant differences in alpha diversity, taxon relative abundance, and SCFA/BCFA levels between groups. A *p*-value ≤ 0.05 was considered statistically significant, while a *p*-value between 0.05 and 0.1 as a trend.

## Results

### Population

Eighty-seven patients aged 1-5 months with grade III-V VUR were enrolled. Sixty-seven (77%) were males, none of them was circumcised. All patients were Caucasian, coming from seven Countries in Europe (Turkey 32, Italy 25, Poland 14, Lithuania 11, Belgium 3, Portugal 1, France 1). The main demographic and clinical characteristics are listed in Table 1.

**Table 1:** Clinical characteristic of enrolled patients.

		**Non-CAP (*n* = 51)**	**CAP (*n* = 36)**	**Tot (*n* = 87)**	***P*-value**	**Odds ratio (95% CI)**
Gender	Males	37 (72.5%)	30 (83.3%)	67 (77%)	0.24	0.53 (0.18, 1.54)
	Females	14 (27.5%)	6 (16.7%)	20 (23%)		
Age (months)	Median (IQR)	3.6 (2.6-4.3)	2.6 (1.7-3.53)	3.1 (2.2-3.9)	**0**. **002**	
Feeding modality	Breastfeeding	30 (60.0%)	28 (80.0%)	58 (68.2%)	0.14	Breastfeeding vs. Formula0.54 (0.09, 3.16)
	Mixed	16 (32.0%)	5 (14.3%)	21 (24.7%)		
	Formula	4 (8%)	2 (5.7%)	6 (7.1%)		
Gestational age	Term	45 (88.2%)	34 (94.4%)	79 (90.8%)	0.53	Term vs. Preterm0.53 (0.10, 2.90)
	Preterm	5 (9.8%)	2 (5.6%)	7 (8%)		
	Postterm	1 (2%)	0	1 (1.1%)		
Probiotics consumption	Yes	8 (15.7%)	4 (11.1%)	12 (13.8%)	0.54	1.49 (0.41, 5.38)
	No	43 (84.3%)	32 (88.9%)	75 (86.2%)		
Mode of delivery	Cesarean section	27 (54.0%)	6 (18.8%)	33 (40.2%)	**0**. **001**	5.09 (1.78, 14.50)
	Spontaneous delivery	23 (46.0%)	26 (81.2%)	49 (59.8%)		
Grade of VUR	Grade III *n* (%)	13 (25.5%)	8 (22.2%)	21 (24.1%)	0.37	Grade III vs. IV/V1.20 (0.44, 3.28)
	Grade IV *n* (%)	18 (35.3%)	18 (50%)	36 (41.4%)		
	Grade V *n* (%)	20 (39.2%)	10 (27.8%)	30 (34.5%)		

*VUR, vesicoureteral reflux; 95% CI, 95% confidence interval. P-values were obtained by Chi squared test for categorical variables and Wilcoxon test for continuous variables (significant values are reported in bold)*.

Regarding CAP exposure, at the time of analysis 51/87 patients had not received any CAP (non-CAP group), while 36/87 patients were taking CAP (CAP group). The median duration of CAP was 47 days (range: 16-140 days). The use of different classes of antibiotics in the CAP group was as follows: amoxicillin or amoxicillin + clavulanic acid 21 (58.3%), trimethoprim 7 (19.4%), oral cephalosporins 6 (16.7%), and nitrofurantoin 2 (5.5%).

### Comparison of Potential Confounding Factors

No short cycles of antibiotics for any acute infection were reported. The groups did not differ by gender, feeding modality, gestational age, and probiotics consumption (Table 1). According to their age between 1 and 5 months, all patients were within the development phase of GM maturation as recently established (4). Delivery mode differed between groups, with a higher proportion of children born by cesarean section in the non-CAP group than in the CAP group (*p* = 0.001, Chi-square test). As mentioned before, all patients were Caucasians and all male were uncircumcised, regardless of the group.

### Gut Microbiota of CAP vs. Non-CAP Infants

**Figure 1:**
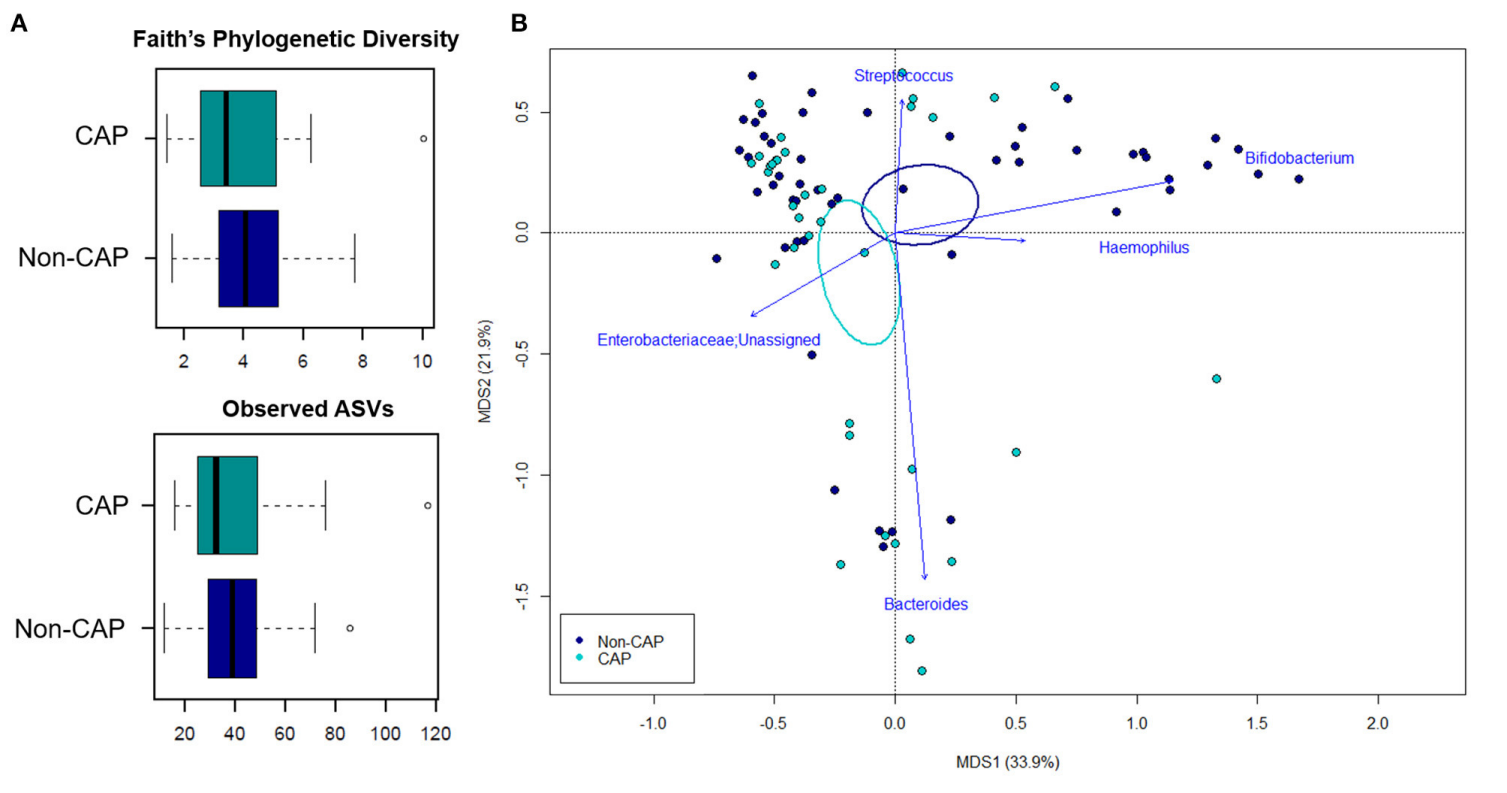
The gut microbiota of infants exposed to CAP segregates from that of non-CAP infants. **(A)** Alpha diversity estimated according to Faith's Phylogenetic Diversity and the number of observed ASVs. No significant differences were found (*p* ≥ 0.2, Wilcoxon test). **(B)** Principal Coordinates Analysis (PCoA) based on weighted UniFrac distances between fecal samples. A significant separation between groups was observed (*p* = 0.015, permutation test with pseudo-F ratios). Ellipses include 95% confidence area based on the standard error of the weighted average of sample coordinates. Bacterial genera with the largest contribution to the ordination space are indicated with blue arrows (*p* ≤ 0.05, permutational correlation test, “envfit” function).

**Figure 2:**
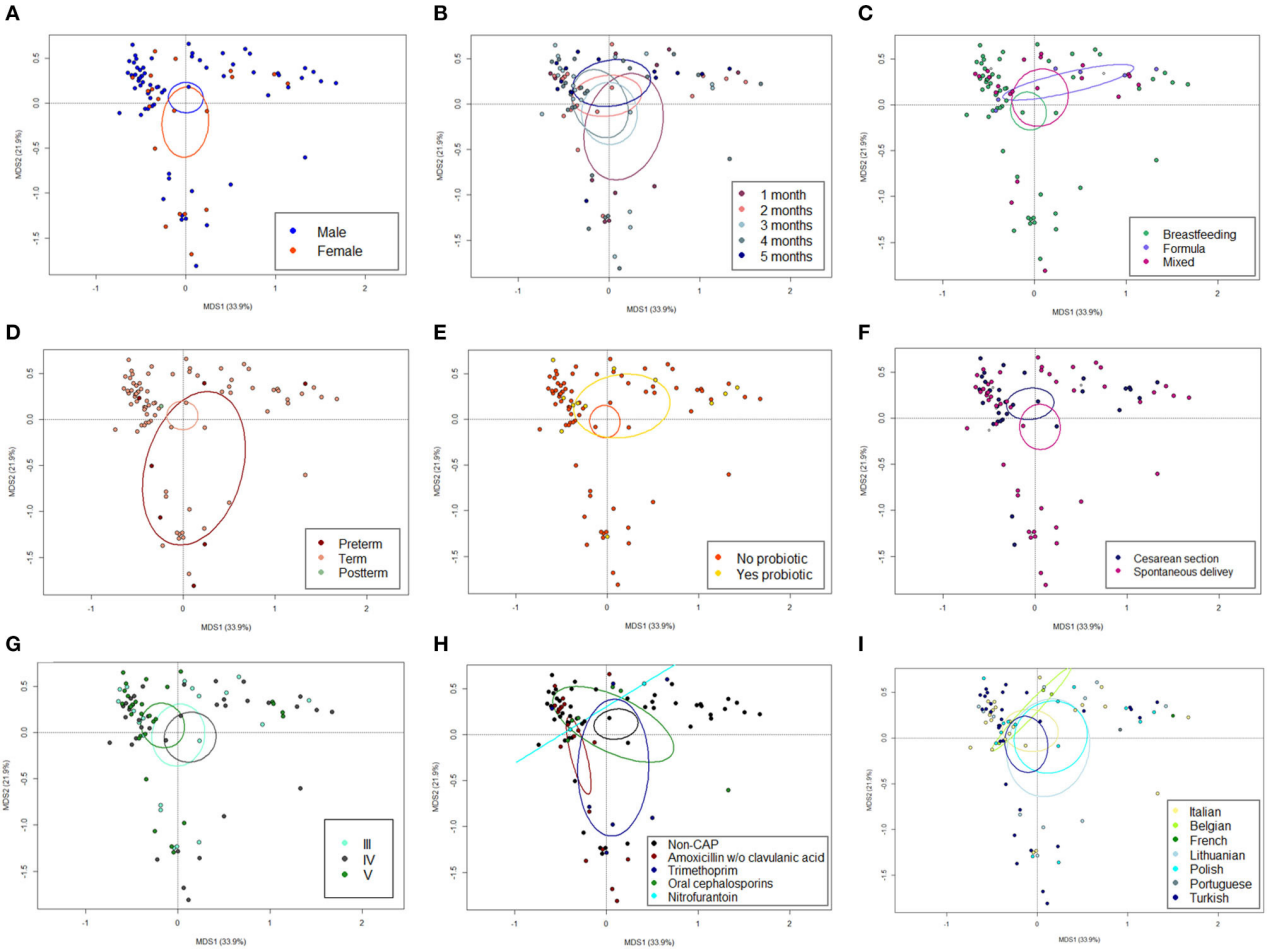
The GM dysbiosis in CAP infants is independent of potential confounding factors. Principal Coordinates Analysis (PCoA) based on weighted UniFrac distances between fecal samples from CAP vs. non-CAP infants, stratified by gender (male vs. female, **A**), age group (1-5 months, **B**), feeding modality (breastfeeding vs. formula vs. mixed, **C**), gestational age (term vs. preterm vs. postterm, **D**), probiotics consumption (yes vs. no, **E**), delivery mode (cesarean section vs. spontaneous delivery, **F**) VUR grade (grade III vs. IV vs. V, **G**), class of antibiotics (amoxicillin or amoxicillin + clavulanic acid, trimethoprim, oral cephalosporins, and nitrofurantoin, **H**) and geographical origin (Italian, Belgian, French, Lithuanian, Polish, Portuguese, and Turkish, **I**). No significant separation was found (*p* ≥ 0.118, permutation test with pseudo-F ratios). Ellipses include 95% confidence area based on the standard error of the weighted average of sample coordinates. See also Figure 1.

The 16S rRNA gene-based sequencing yielded a total of 908,524 high-quality reads, with an average of 10,443 ± 2,920 sequences per sample, binned into 3,075 ASVs. No differences in alpha diversity were observed between CAP and non-CAP patients (*p* ≥ 0.2, Wilcoxon test) (Figure 1A). Principal Coordinates Analysis (PCoA) of inter-individual variation, based on weighted UniFrac distances, revealed significant separation between groups (*p* = 0.015, permutation test with pseudo-F ratios- Figure 1B). By contrast, no significant differences were detected according to the unweighted UniFrac metrics (Supplementary Figure 2). Interestingly, no separation was observed with regards to any of the potential confounders evaluated, such as gender, age group, feeding modality, gestational age, probiotics consumption, delivery mode, VUR grade, class of antibiotics, and geographical origin (Figure 2).

**Figure 3:**
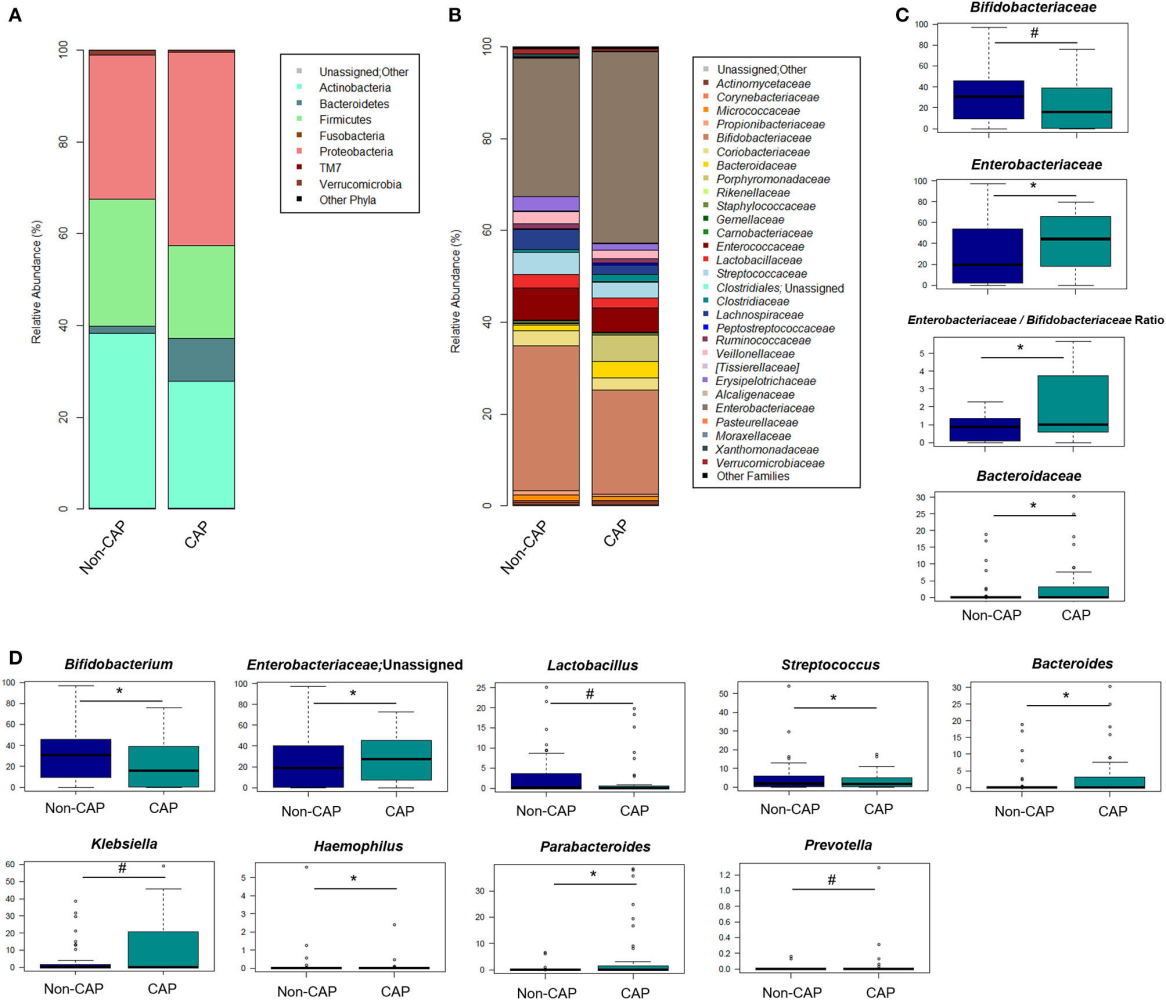
Gut microbiota structure in CAP vs. non-CAP infants. **(A,B)** Bar plots representing the relative abundance of the major phyla **(A)** and families **(B)** in the GM of CAP and non-CAP infants. **(C,D)** Box plots showing the relative abundance distribution of bacterial families **(C)** and genera **(D)** with a significant difference (*p* ≤ 0.05, Wilcoxon test; asterisk) or a trend (0.05 < *p* ≤ 0.1; hashtag) between the two groups. Only taxa with relative abundance >0.1% in at least two samples were considered.

The GM of CAP and non-CAP infants was dominated by the phyla Actinobacteria, Proteobacteria, and Firmicutes. However, CAP patients were significantly enriched in Bacteroidetes (*p* = 0.03, Wilcoxon test) (Figure 3A). Although the family-level GM layout of both groups was generally characterized by a preponderance of *Bifidobacteriaceae* and *Enterobacteriaceae* (Figure 3B), significant differences emerged. Specifically, compared to non-CAP infants, those exposed to CAP showed significant enrichment in *Bacteroidaceae* (*p* = 0.03) and *Enterobacteriaceae* (*p* = 0.05), as well as a trend toward reduced proportions of *Bifidobacteriaceae* (*p* = 0.09). As expected, the ratio of *Enterobacteriaceae* to *Bifidobacteriaceae*, a validated index to measure intestinal health and microbial colonization resistance, with a higher value reflecting a compromised ecosystem (20), was significantly higher in CAP group than in the non-CAP group (*p* = 0.05) (Figure 3C).

In order to identify the genera responsible for the separation between CAP and non-CAP infants, relative abundance vectors with a statistically significant contribution were overlaid onto the weighted UniFrac ordination space of Figure 1B (*p* ≤ 0.05, “envfit” function). *Bacteroides* and unclassified genera of *Enterobacteriaceae* were associated with CAP, while *Bifidobacterium, Haemophilus*, and *Streptococcus* were associated with the non-CAP group (*p* ≤ 0.05) (Figure 3D). Furthermore, the GM profile of CAP patients was characterized by increased proportions of *Parabacteroides* (*p* = 0.05), as well as by a tendency toward increased amounts of *Klebsiella* and *Prevotella* and reduced amounts of *Lactobacillus* (*p* ≤ 0.1) (Figure 3D).

Most of the differences in GM diversity and composition between CAP and non-CAP infants persisted or tended to persist even after excluding patients who were receiving probiotics (Supplementary Figure 3), which further reinforces that GM dysbiosis in CAP infants is independent of potential confounding factors, such as intake of probiotics.

### Fecal Levels of SCFAs and BCFAs

SCFA and BCFA fecal levels were determined on 52/87 infants, of whom 29 were not taking prophylaxis whereas 23 were under CAP. No significant differences were found between groups in the total amount of SCFAs, as well as in the individual amounts of acetic, butyric, propionic, and valeric acid (*p* > 0.05). Similarly, total BCFA levels and those of isobutyric and isovaleric acid were not different according to CAP exposure (*p* > 0.05) (Supplementary Figure 4).

## Discussion

GM maturation is a fine process shaped by various environmental factors (21). The first months of life are a critical time window during which GM-host cross-talk lays the foundations for future health (8). Concerns are rising about the influence of antibiotics on the highly dynamic infantile GM (7).

Infants with VUR often receive prolonged CAP to prevent UTIs, but the potential benefits have to be balanced against long-term side effects.

Comparing infants with VUR previously treated with CAP and those naïve, our study showed an altered GM composition even after a short (47 days) exposure to a sub-therapeutic dosage of antibiotics. GM changes involved the loss of typical features of the eubiotic infant-type ecosystem, with decreased proportions of *Bifidobacterium*, a keystone taxon in the infant GM, which dominates the ecosystem at least until breastfeeding has ceasesed (4). *Bifidobacterium* spp. are indeed able to thrive on human milk oligosaccharides and are associated with many health-promoting functions, including the development of the immune system (22). Conversely, compared to non-CAP patients, the GM of CAP infants was enriched in *Bacteroides* and *Parabacteroides*, and showed a trend toward increased proportions of *Prevotella*. According to the TEDDY study, which followed the GM trajectory in 903 children from 3 to 46 months of age (4), *Bacteroides* is associated with increased GM diversity and faster maturation during early life, regardless of the birth mode. Analogously, *Prevotella* is another well-known marker of the adult GM, although associated with host variables, including diet and geography (17). It is worth noting that three enterotype-like clusters dominated by *Bacteroides, Prevotella*, and *Bifidobacterium*, have recently been detected in the microbiota of school-aged children and suggested to represent stratified developmental trends of GM toward the adult configuration, with the *Bifidobacterium* enterotype being the less mature (23). It is therefore tempting to speculate that CAP exposure may induce a non-eubiotic, accelerated maturation of the infant GM toward an adult-like profile.

Moreover, the GM of CAP patients was enriched in *Enterobacteriaceae*, specifically *Klebsiella* (albeit with poor significance), a well-known clinically relevant nosocomial pathogen, associated with systemic infections, including antibiotic-resistant UTIs (24). In addition to indicating ineffectiveness of prophylaxis in controlling its spread, the high amounts of *Klebsiella* in the CAP group could represent a non-negligible risk factor for the development of more difficult-to-treat UTIs in this highly vulnerable population.

While several potential confounders such as feeding modality, age, gender, VUR grade, class of antibiotics, and geographical origin, did not differ between groups, a higher number of non-CAP infants had undergone cesarean section. Although this may have partially biased our results, particularly regarding *Bacteroides*, which has sometimes been found enriched in infants born vaginally (25), we did not find a significant impact of the delivery mode on GM composition, when we performed a sub-analysis according to all assessed confounding factors. Similarly, we did not observe a significant separation in the GM structures by intake of probiotics and even excluding patients who were receiving them, we obtained the same diversity findings and recovered the main discriminating taxa (although sometimes only trends due to the decreased sample size). This confirms the specific impact of antibiotics on CAP-related dysbiosis.

Previous reports have analyzed the impact of antibiotics on infantile GM, but in different settings, with less homogeneous populations. In 2014, a study on preterm infants demonstrated decreased diversity after 5-7 days of empiric antibiotic therapy with either ampicillin or gentamicin, resulting in compositional imbalances, such as increased *Enterobacter* (26). In 2016, Bokulich and coworkers showed in 43 healthy infants that prenatal and postnatal antibiotic exposure to different classes and doses of antibiotics delays GM maturation and suppresses important pathobionts (8). A study on 39 children aged 0-3 years observed lower diversity and a peak of resistance genes after multiple courses (9–15) of full-dose antibiotic therapy (27). Persistent GM differences, not prevented by probiotics consumption, were demonstrated in 149 newborns exposed to perinatal (prenatal and/or postnatal) antibiotics compared to controls (28). On the other hand, Akagawa et al. showed a non-significant effect of long-term CAP (up to 6 months) on the GM of 35 patients younger than 3 years (29). However, CAP was administered to older children who had previously received antibiotic treatment for UTIs, making any comparisons with our data meaningless.

Our analysis sheds further light on the impact of antibiotics on GM in infants, revealing that even a short exposure to a sub-therapeutic dose of antibiotics in the absence of previous infections, results in detectable GM modification, in such a delicate moment for GM maturation. The observed deviation probably implies an impairment of the barrier effect, as supported by the almost significant increase in *Klebsiella*. Although the CAP-associated dysbiosis did not lead to imbalanced SCFA and BCFA production, a delayed effect in the following months/years of life cannot be excluded, when a more fermentative ecosystem is being established. Indeed, it has been demonstrated that early deviations from a eubiotic GM layout can provide long-lasting pathophysiological problems, as a result of a compromised GM-host dialogue that fails in GM-dependent immunological and metabolic programming (6, 9).

The GM dysbiosis observed after a short exposure to CAP points to potentially even deeper alterations that may be caused by the extended, typically 2-year courses commonly prescribed to VUR patients. Some authors suggest that GM disruption during a critical age contributes to an increased risk of diseases such as asthma, obesity, and allergies (6, 7, 30, 31). Two different studies including over 23,000 children proved that antibiotic exposure before 6 months is linked to increased body mass later in infancy (32, 33). This association is believed to be related to GM modifications (8). Mouse models have provided clear evidence for a role of the GM in growth regulation, metabolism and fat storage (9, 34). Furthermore, GM dysbiosis has been linked to cardiovascular morbidity in children with chronic kidney disease (35). Finally, antibiotic pressure increases drug-resistance genes in GM microorganisms (“resistome”), with potential horizontal transfer (7).

The study has some limitations. The relatively small sample size may have underestimated between-group differences, with particular reference to *Klebsiella, Prevotella*, and *Lactobacillus*. Nonetheless, the number of patients enrolled is comparable to the available literature and the other observed differences in the GM composition are supported by the statistical analysis. In addition, the distribution of patients born by cesarean section was unbalanced, CAP consisted of different types of antibiotics and some patients were receiving probiotics. These factors (birth mode, antibiotic type, and probiotic use) are known to be associated with GM variation. Nonetheless, the respective sub-analyses showed no significant impact on the GM structure, as for all the other potential confounders considered, suggesting that the observed changes are likely attributable to prophylaxis *per se*.

Given the observational and cross-sectional design, further prospective studies in larger cohorts will be needed to confirm the persistence of GM alterations and their impact on children health, later in life. In this regard, the ongoing randomized controlled PREDICT trial, prospectively monitoring the GM trajectories of CAP-exposed and unexposed infants together with clinical information regarding obesity, allergies, or multidrug-resistant infections, is expected to define the real long-term impact of CAP.

## Data Availability Statement

The datasets presented in this study can be found in online repositories. The names of the repository/repositories and accession number(s) can be found below: https://www.ncbi.nlm.nih.gov/bioproject/706153, PRJNA706153.

## Ethics Statement

The study involving human participants was reviewed and approved by Comitato etico Milano Area 2. Written informed consent to participate in this study was provided by the participants' legal guardian/next of kin.

## Author Contributions

WM, ST, JF, GM, and MC designed the study, performed samples analysis, data interpretation, statistical analysis and figures, drafted the initial manuscript, and reviewed and revised the manuscript. FDA, JS, and IA performed samples analysis, data interpretation, statistical analysis and figures, drafted the initial manuscript, and reviewed and revised the manuscript. EB, FY, AJ, MP, AZ, FB, DD, DM, GK, CL, and KT-J collected biological samples, provided clinical data, reviewed the findings, and contributed to the interpretation. OM and FS contributed to data interpretation and critically reviewed the manuscript. All authors participated in manuscript revision and approved the final version.

## Conflict of Interest

The authors declare that the research was conducted in the absence of any commercial or financial relationships that could be construed as a potential conflict of interest.

## Supplementary Material

Supplementary Figure 1 Flow diagram of screened and enrolled population.

Supplementary Figure 2 Principal Coordinates Analysis (PCoA) based on unweighted UniFrac distances between fecal samples from CAP vs. non-CAP infants. No significant separation between groups was found (*p* = 0.346, permutation test with pseudo-F ratios). Ellipses include 95% confidence area based on the standard error of the weighted average of sample coordinates.

Supplementary Figure 3 The GM dybsiosis in CAP infants is independent of probiotics intake. **(A)** Alpha diversity estimated according to Faith's Phylogenetic Diversity and the number of observed ASVs. No significant differences were found (*p* ≥ 0.1, Wilcoxon test). **(B)** Principal Coordinates Analysis (PCoA) based on weighted UniFrac distances between fecal samples. A significant separation between groups was observed (*p* = 0.05, permutation test with pseudo-F ratios). Ellipses include 95% confidence area based on the standard error of the weighted average of sample coordinates. Bacterial genera with the largest contribution to the ordination space are indicated with blue arrows (*p* ≤ 0.05, permutational correlation test, “envfit” function). **(C)** Boxplots showing the relative abundance distribution of bacterial taxa that tended to be differentially represented between the two groups (0.05 < *p* ≤ 0.1, Wilcoxon test). Only taxa with relative abundance > 0.1% in at least two samples were considered. For this analysis, only patients who were not receiving probiotics were included (i.e., 32 vs. 43 in the CAP vs. non-CAP group). See also Table 1.

Supplementary Figure 4 Fecal levels of SCFAs and BCFAs in infants exposed or not to CAP. Boxplots showing the absolute amount distribution for SCFAs (acetic, propionic, butyric, and valeric acids) and BCFAs (isobutyric and isovaleric acids) measured in μmol/g. No significant differences were found (*p* > 0.05, Wilcoxon test).
