# Supplementary figures and images for: Low-Dose Antibiotic Prophylaxis Induces Rapid Modifications of the Gut Microbiota in Infants With Vesicoureteral Reflux
Source: Front Pediatr. 2021 Jun 17;9:674716. doi: 10.3389/fped.2021.674716 (PMC8247656; doi:10.3389/fped.2021.674716)

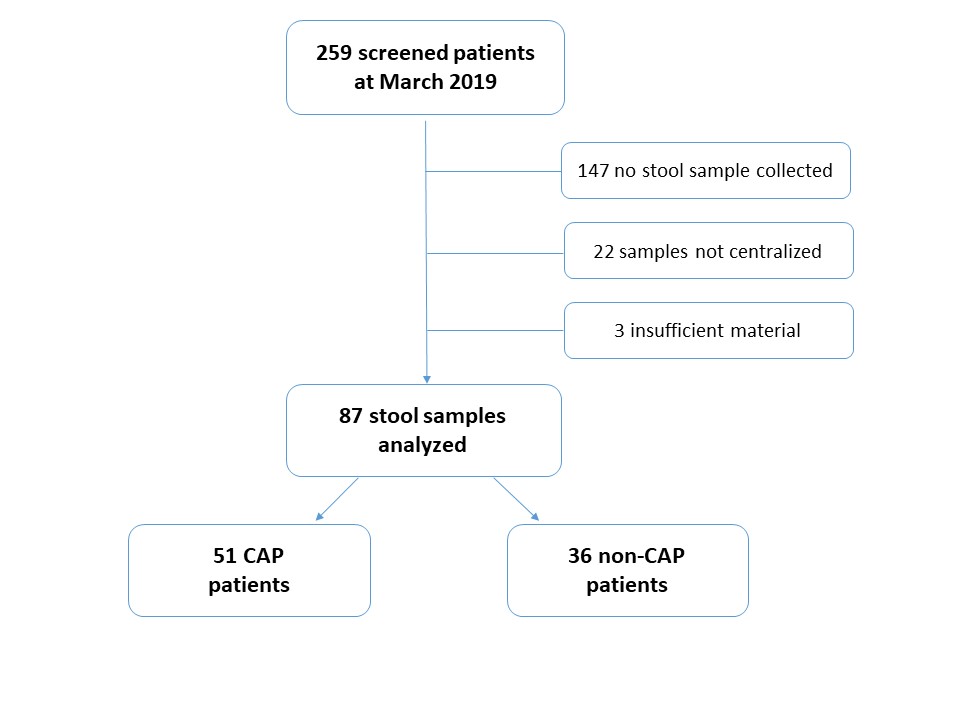

Supplement: Supplementary Figure 1 — Flow diagram of screened and enrolled population. [file Image_1.JPEG]

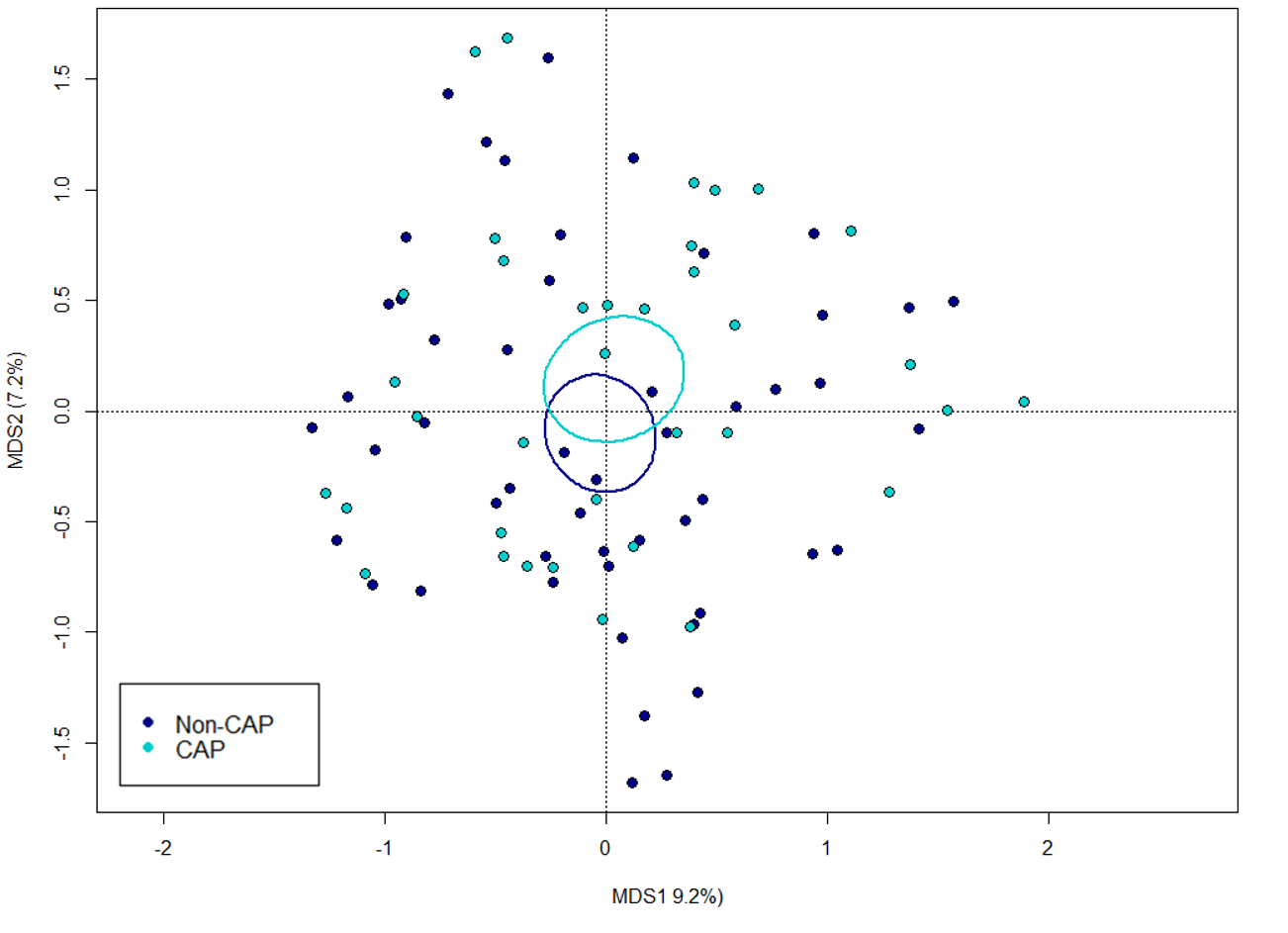

Supplement: Supplementary Figure 2 — Principal Coordinates Analysis (PCoA) based on unweighted UniFrac distances between fecal samples from CAP vs. non-CAP infants. No significant separation between groups was found (p = 0.346, permutation test with pseudo-F ratios). Ellipses include 95% confidence area based on the standard error of the weighted average of sample coordinates. [file Image_2.TIF]

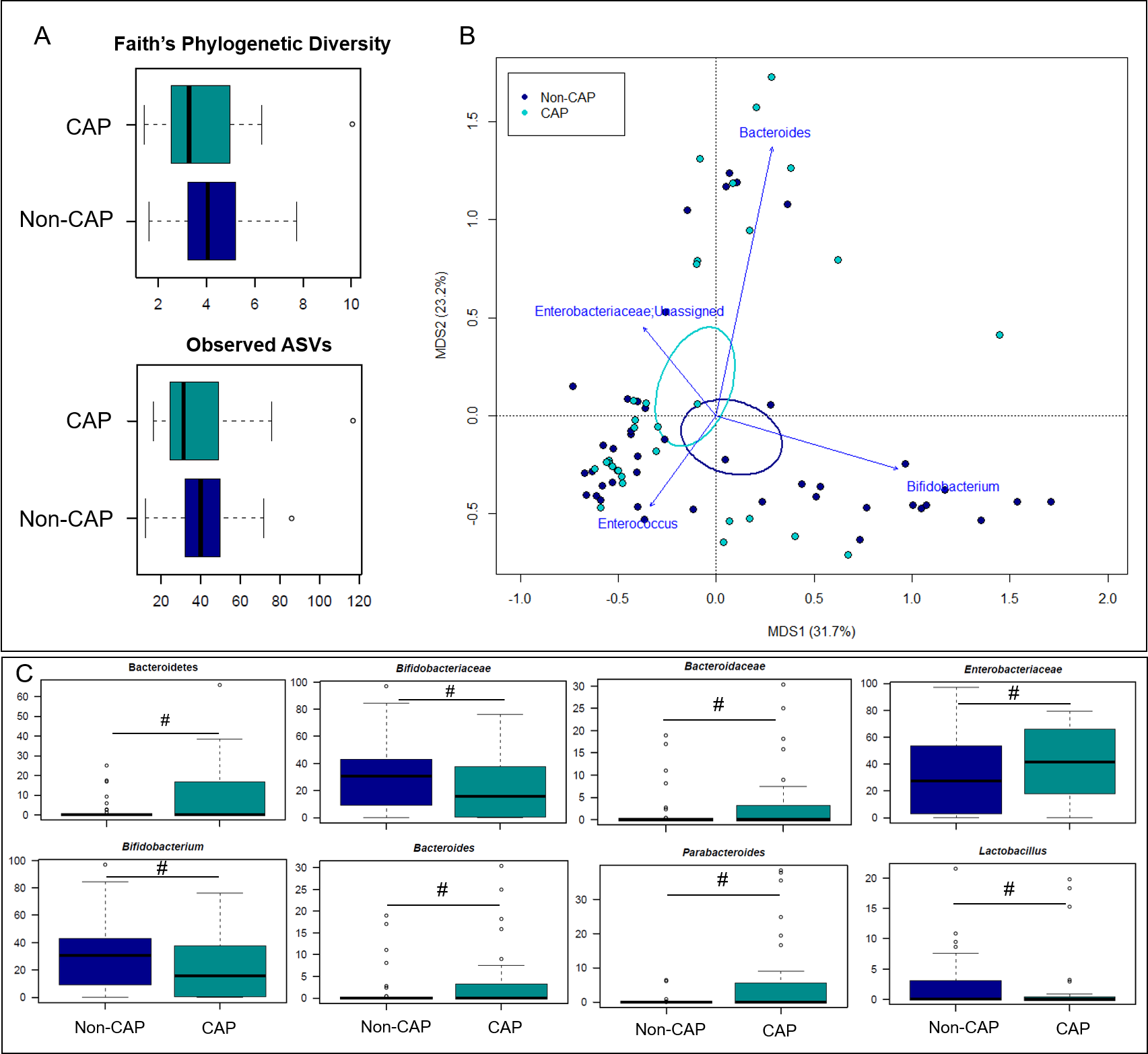

Supplement: Supplementary Figure 3 — The GM dybsiosis in CAP infants is independent of probiotics intake. (A) Alpha diversity estimated according to Faith's Phylogenetic Diversity and the number of observed ASVs. No significant differences were found (p ≥ 0.1, Wilcoxon test). (B) Principal Coordinates Analysis (PCoA) based on weighted UniFrac distances between fecal samples. A significant separation between groups was observed (p = 0.05, permutation test with pseudo-F ratios). Ellipses include 95% confidence area based on the standard error of the weighted average of sample coordinates. Bacterial genera with the largest contribution to the ordination space are indicated with blue arrows (p ≤ 0.05, permutational correlation test, “envfit” function). (C) Boxplots showing the relative abundance distribution of bacterial taxa that tended to be differentially represented between the two groups (0.05 < p ≤ 0.1, Wilcoxon test). Only taxa with relative abundance > 0.1% in at least two samples were considered. For this analysis, only patients who were not receiving probiotics were included (i.e., 32 vs. 43 in the CAP vs. non-CAP group). See also Table 1. [file Image_3.TIF]

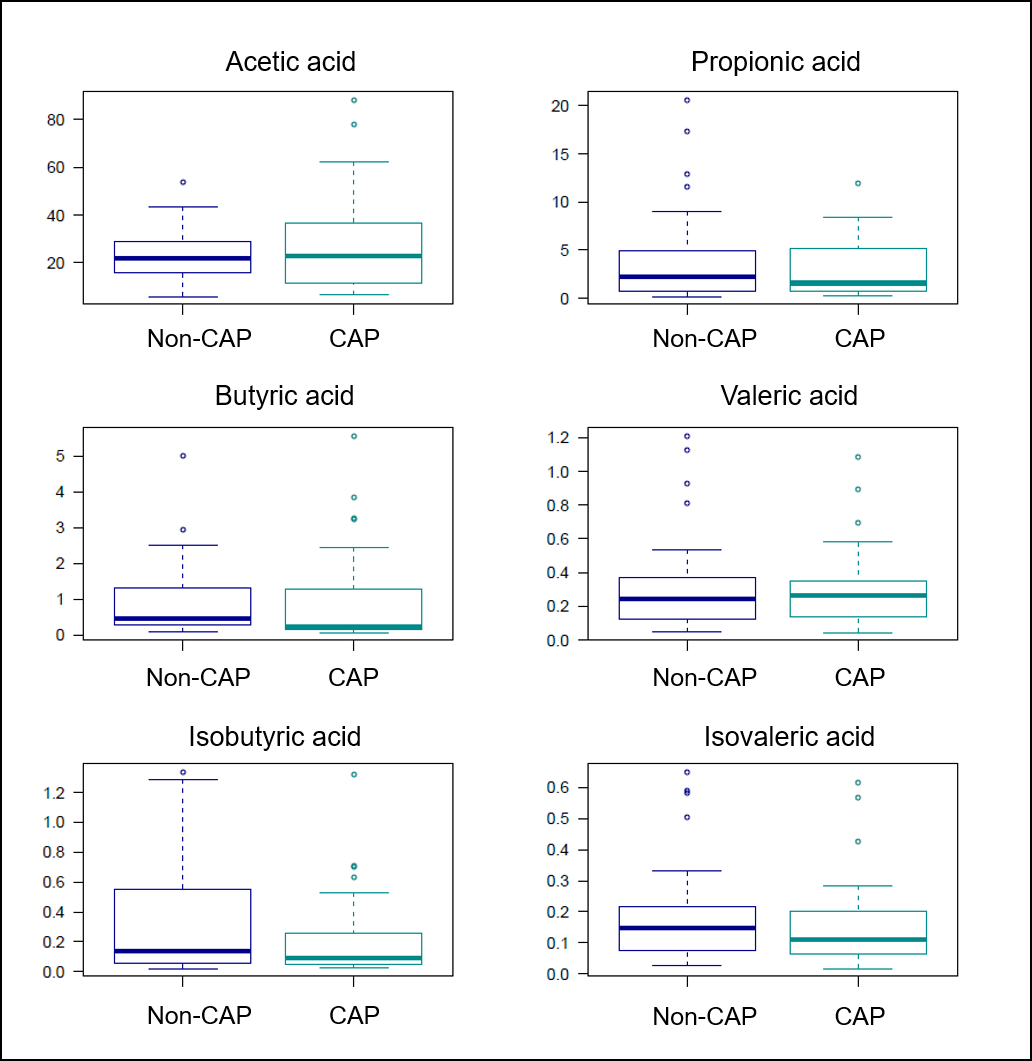

Supplement: Supplementary Figure 4 — Fecal levels of SCFAs and BCFAs in infants exposed or not to CAP. Boxplots showing the absolute amount distribution for SCFAs (acetic, propionic, butyric, and valeric acids) and BCFAs (isobutyric and isovaleric acids) measured in μmol/g. No significant differences were found (p > 0.05, Wilcoxon test). [file Image_4.TIF]
